# Supplementary material for: Assembly of CRISPR ribonucleoproteins with biotinylated oligonucleotides via an RNA aptamer for precise gene editing
Source: Nat Commun. 2017 Nov 23;8:1711. doi: 10.1038/s41467-017-01875-9 (PMC5700129; doi:10.1038/s41467-017-01875-9)
Supplement: Supplementary file 1 — Supplementary Information [file 41467_2017_1875_MOESM1_ESM.pdf]

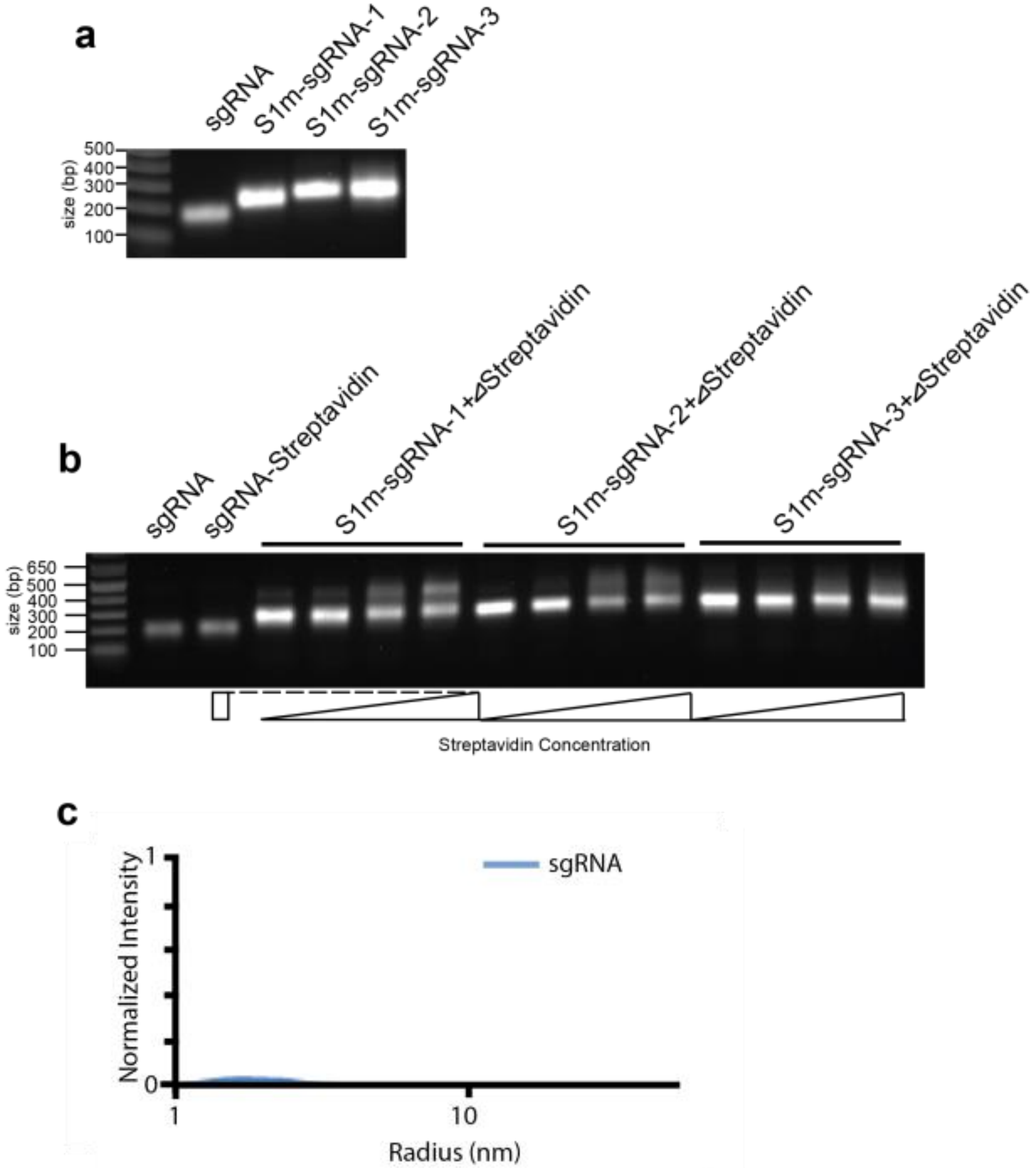

Supplementary Figure 1. Creation and *in vitro* testing of S1m-sgRNA.

a. *In vitro* transcription of S1m-sgRNAs compared to standard sgRNAs. S1m-sgRNAs are larger than sgRNAs due to the insertion of S1m stem loop.

b. *In vitro* complexes of sgRNAs and streptavidin. Lane 1: sgRNA. Lane 2: sgRNA-streptavidin. Addition of streptavidin did not shift the electrophoretic front. Lanes 3-6, 7-10, 11-14: Progressive ratios of each S1m-sgRNA streptavidin (0, 0.1, 1, and 10 molar equivalents of streptavidin). As streptavidin concentration was increased the electrophoretic front of S1m-sgRNAs was slowed. The presence of several bands may be due to multiple S1m-sgRNAs binding to a single streptavidin.

c. DLS trace of free sgRNA alone in solution. Nucleic acids are not robustly detected by this DLS instrument.

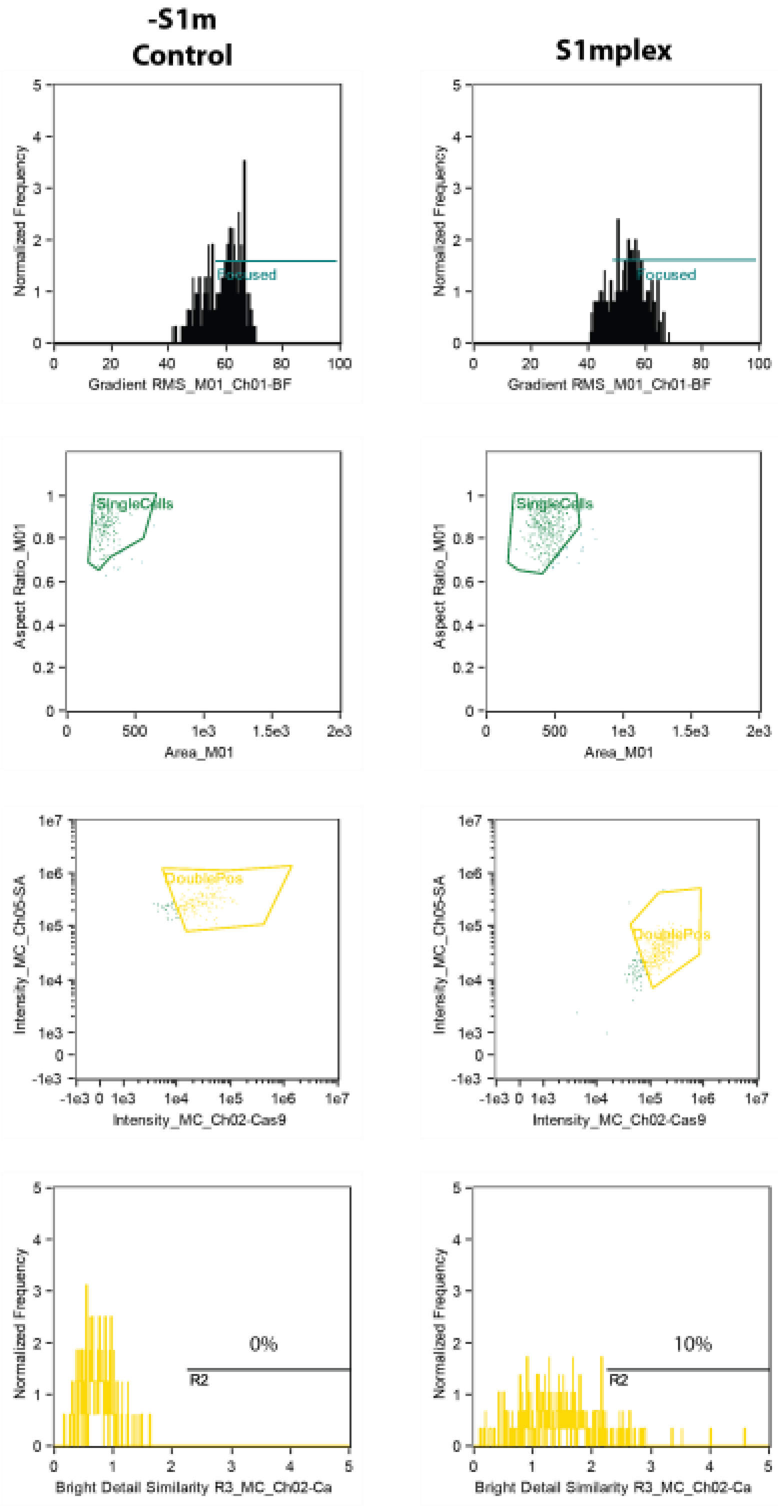

Supplementary Figure 2. Multispectral imaging flow cytometric analysis.
Gating strategy used by multispectral imaging flow cytometry analysis for colocalization of Cas9 and streptavidin. hPSCs were first gated to be in focus. Next, the aspect ratio and area measured from brightfield (Ch1) images were plotted against each other to identify single cells. Then, intensities of Cas9 (Ch2) and streptavidin (Ch5) were plotted to identify double positive cells. Finally, IDEAS software wizard identified cells with high similarity between Ch2 and Ch5 (Bright Detail Similarity).

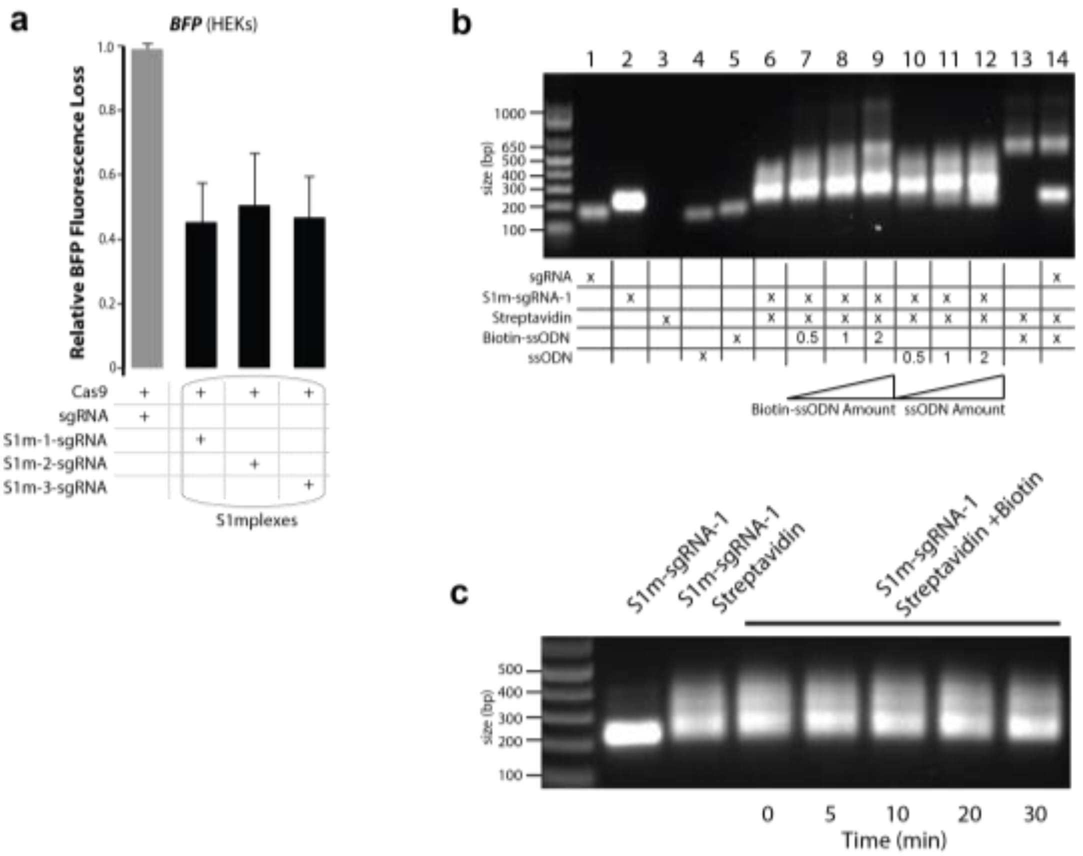

Supplementary Figure 3. Gene editing activity and in vitro assembly of S1m-sgRNAs with streptavidin and ssODNs.

a. Knockout of integrated BFP fluorescence in human embryonic kidney (HEK) cells. When transfected together with a plasmid encoding *Sp*.Cas9, S1m-sgRNAs induced ~50% the level of NHEJ as sgRNA as measured by the loss of fluorescence five days post transfection (error bars±1 s.d., n=3 biological replicates).

b. *In vitro* tertiary complexes of S1m-sgRNA-1, streptavidin, and ssODN. Lanes 1-5: Components of ssODN-S1mplex particles ran individually. Lanes 6-9: complexes of S1m-sgRNA-1, streptavidin, and biotinylated ssODNs. Numbers represent relative stoichiometry between components ran on gel. Major bands showing the complex of all three components can be seen. Lanes 10-12: complexes of S1m-sgRNA-1, streptavidin, and free ssODNs. ssODNs do not interfere with the binary S1m-sgRNA-1/streptavidin complex. Lane 13-14: complexes of streptavidin and biotin-ssODNs, with free sgRNAs. None of the typical S1m-sgRNA-1-streptavidin complexes can be seen in this lane.

c. Competition of biotin with S1m-sgRNA-1 after binding to streptavidin protein. 4-fold excess biotin was added to S1m-sgRNA-1-streptavidin complexes and incubated for 0, 5, 10, 20, and 30 minutes. No significant change was seen even after 30 minutes of competition.

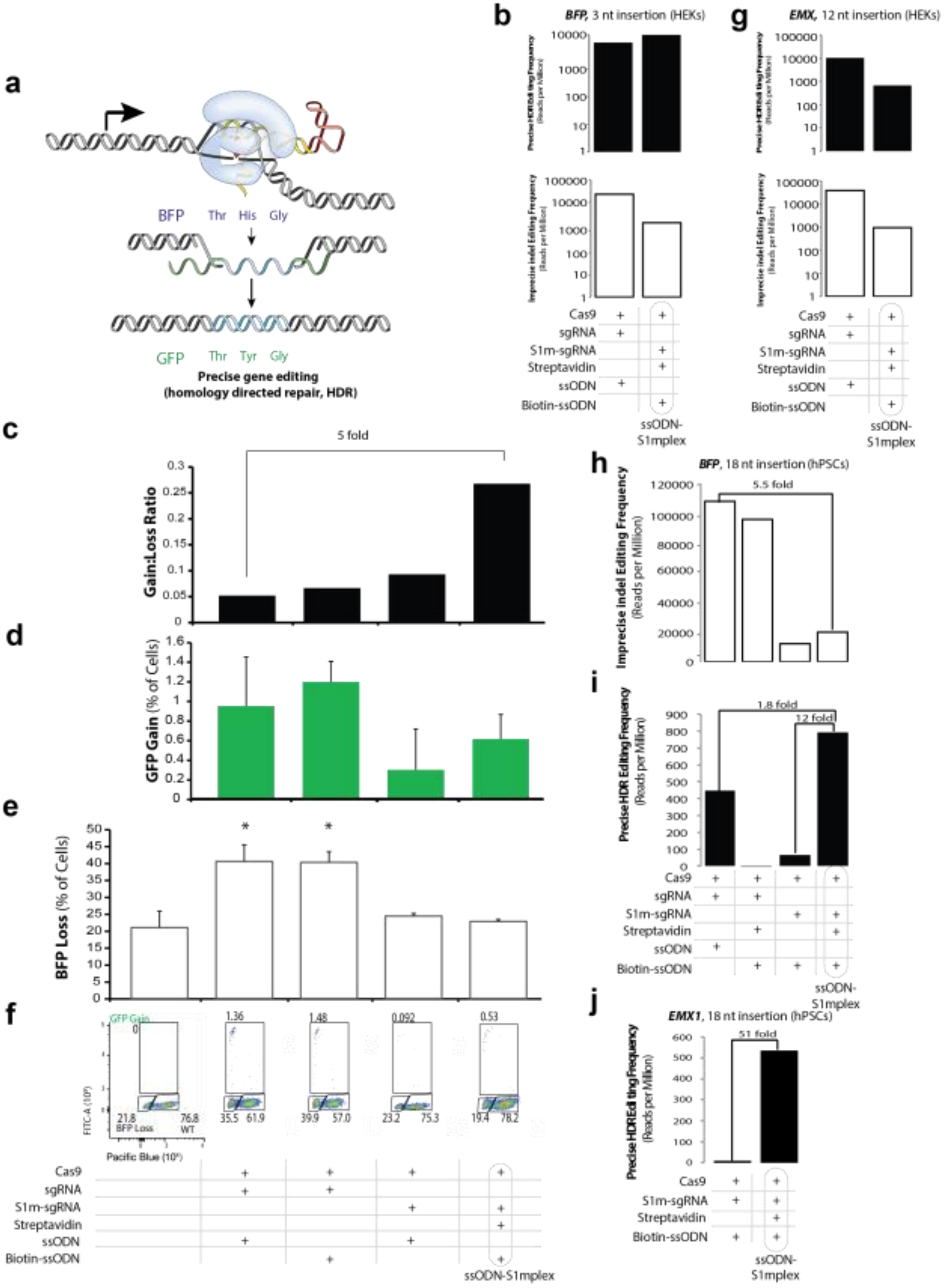
a
BFP Thr His Gly
GFP Thr Tyr Gly
Precise gene editing
(homology directed repair, HDR)
b
BFP, 3 nt insertion (HEKs)
g
EMX, 12 nt insertion (HEKs)
Cas9
sgRNA
S1m-sgRNA
Streptavidin
ssODN
Biotin-ssODN
ssODN-S1mplex
c
5 fold
Gain:Loss Ratio
d
GFP Gain (% of Cells)
e
BFP Loss (% of Cells)
f
GFP Gain
BFP Loss
WT
Pacific Blue
FITC-A
h
BFP, 18 nt insertion (hPSCs)
5.5 fold
Imprecise Indel Editing Frequency
(Reads per Million)
i
1.8 fold
12 fold
Precise HDR Editing Frequency
(Reads per Million)
j
EMX1, 18 nt insertion (hPSCs)
51 fold

Supplementary Figure 4. Increased ratio of precise to imprecise editing using ssODN-S1mplexes.
a. Schematic showing HDR conversion of *BFP* locus to GFP using ssODN-S1mplex particles.
b. *Top:* Number of precise insertion reads at *BFP* locus. S1mplexes had higher levels of insertion than sgRNA RNPs. *Bottom:* Number of indel reads at *BFP* locus. S1mplexes had lower levels of imprecise edits than sgRNA RNPs.
c. Ratio of GFP gain to BFP loss using four different editing methods. ssODN-S1mplexes had 5-fold higher ratio of gain:loss than free ssODN and gRNA.
d. ssODN-S1mplexes had similar levels of GFP gain when compared to free ssODN and gRNA. Using S1m-sgRNAs with free ssODN had low levels of HDR (error bars ±1 s.d., n=3 biological replicates).
e. Standard sgRNAs caused elevated levels of BFP loss when compared to the control cell lines. S1m-sgRNAs did not result in significant levels of NHEJ (*$p<0.05$, Student's two-tailed t-test, error bars ±1 s.d., n=3 biological replicates).
f. Representative flow cytometry plots from all conditions.
g. *Top:* Number of precise insertion reads at *EMX1* locus. S1mplexes had lower levels of insertion than sgRNA RNPs. *Bottom:* Number of indel reads at *EMX1* locus. S1mplexes had significantly lower levels of imprecise edits than sgRNA RNPs.
h. ssODN-S1mplexes decrease the frequency of imprecise NHEJ editing events 5.5-fold compared to RNPs.
i. Frequency of precise insertions in *BFP* locus measured by deep sequencing. ssODN-S1mplexes had 1.8 times more insertions than standard sgRNA RNPs and 12 times more insertions than S1m-sgRNAs without complexed ssODNs due to lack of streptavidin. When sgRNAs were transfected with biotin-ssODNs there were nearly no precise insertions.
j. Frequency of precise insertions at *EMX1* locus. Addition of streptavidin to bind S1m-sgRNAs and biotin-ssODN resulted in a 51-fold increase in the frequency of precise editing reads.

See also Supplementary Table 8 for absolute levels of editing.

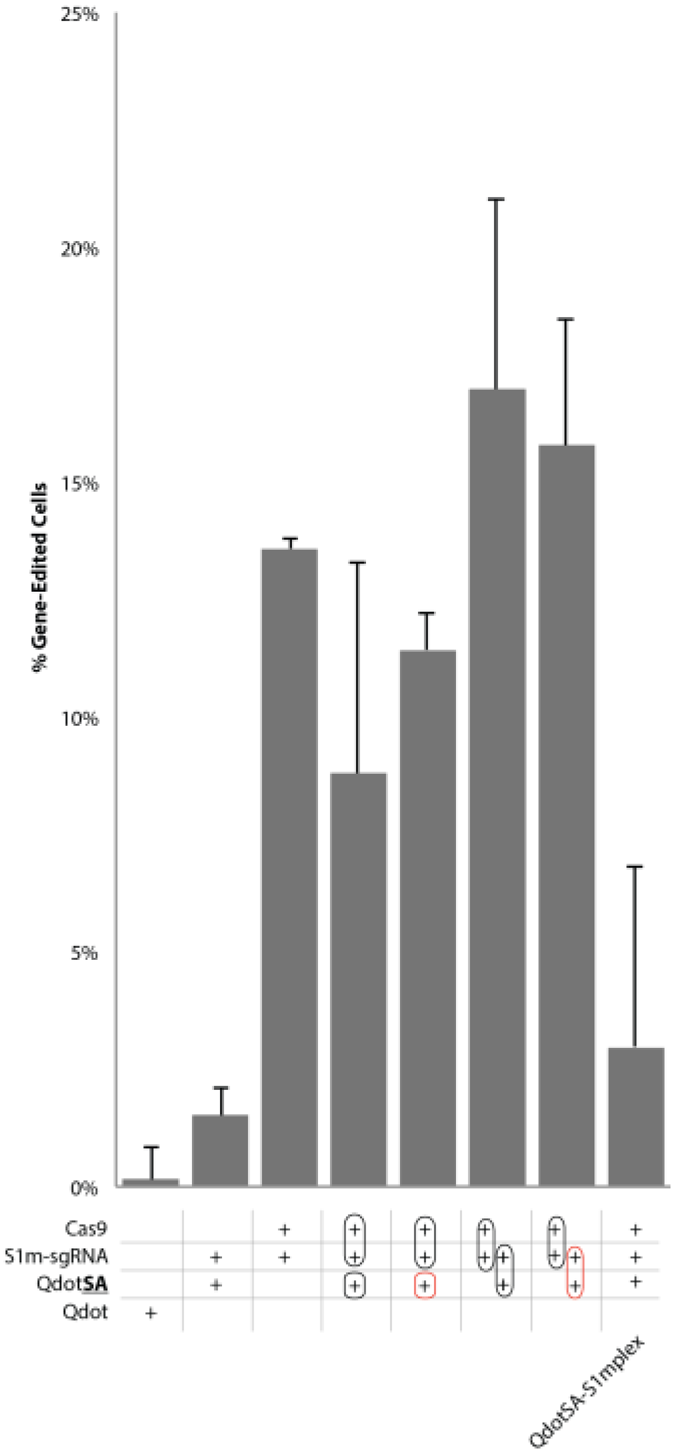

Supplementary Figure 5. Gene-editing using various combinations of components with QdotSA.
Conjugation of S1mplexes to QdotSA significantly lowers gene editing efficiency with H2B-mCherry HEKs. Editing efficiency is lower even if QdotSA is transfected separately from the S1mplexes without complexation. Bounding boxes denote separate transfections and red bounding boxes denote separate transfection of reagents in the red box 5 hours later. Immediate application of the QdotSA can moderately interfere with the activity of the RNP, but these interference effects are abrogated if QdotSA is added 5 hours later. All RNP activity is abrogated by assembly with the QdotSA (last column) (error bars ±1 s.d., n=3 technical replicates).

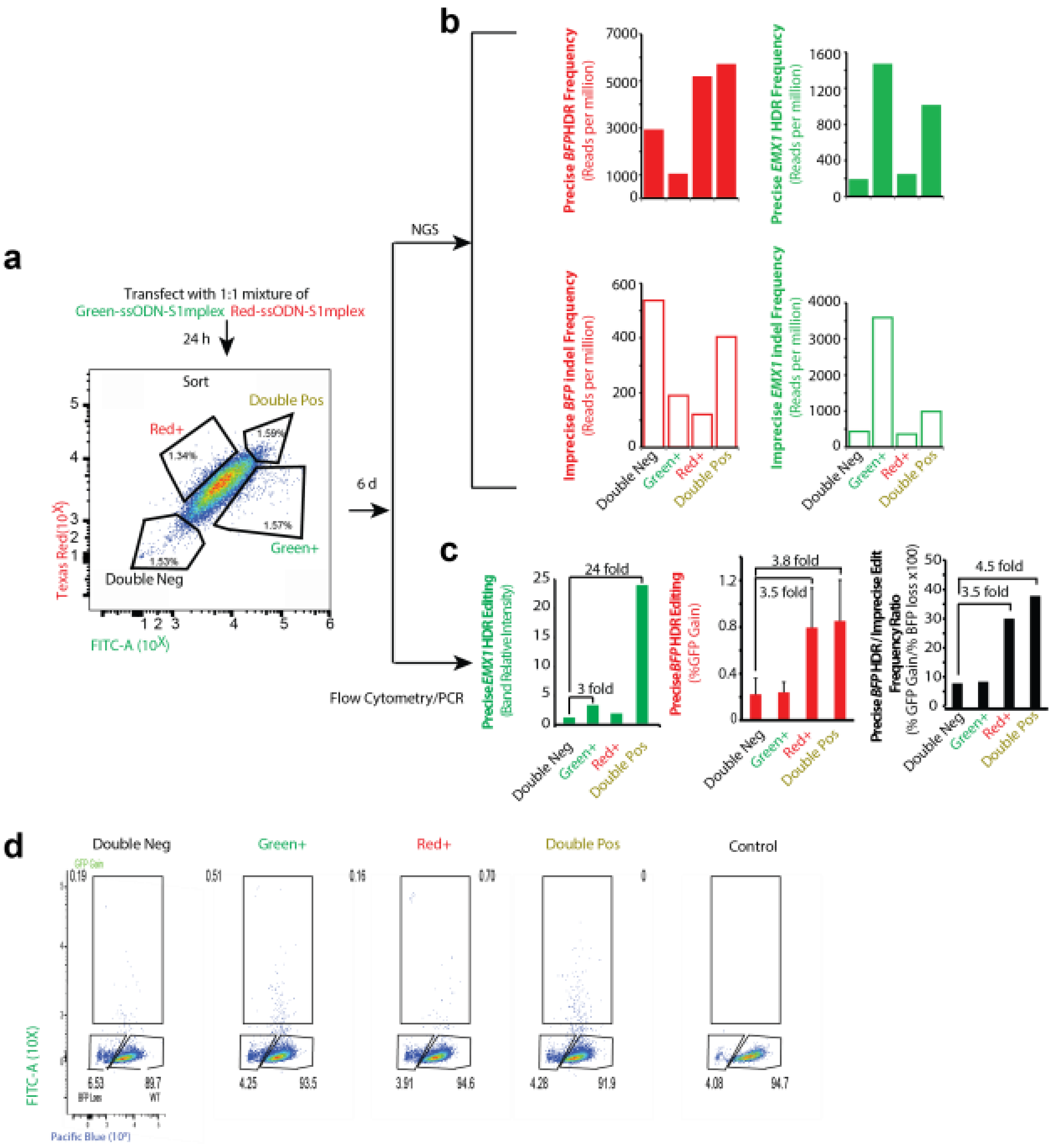

Supplementary Figure 6. Multiplexed editing with fluorescent ssODN-S1mplexes.

a. Flow cytometry plot of fluorescent S1mplexes 24 hours post transfection, HEKs were sorted into populations that were positive for either fluorophore, both or neither. Analysis was done 6 days post sorting.

b. Precise and imprecise editing frequency measured by deep sequencing for all four sorted populations. Precise editing was enriched in populations specific for an ssODN-S1mplex. Imprecise editing was not uniformly higher in sorted populations for a specific sgRNA.

c. Precise and imprecise editing measured by conventional methods for all four sorted populations. Populations specific for editing at a single locus as well as the double positive population were enriched for edits at that locus and not the other (error bars ±1 s.d., n=3 biological replicates).

d. Representative flow cytometry plots related to Figure 6d and parts a-c above.

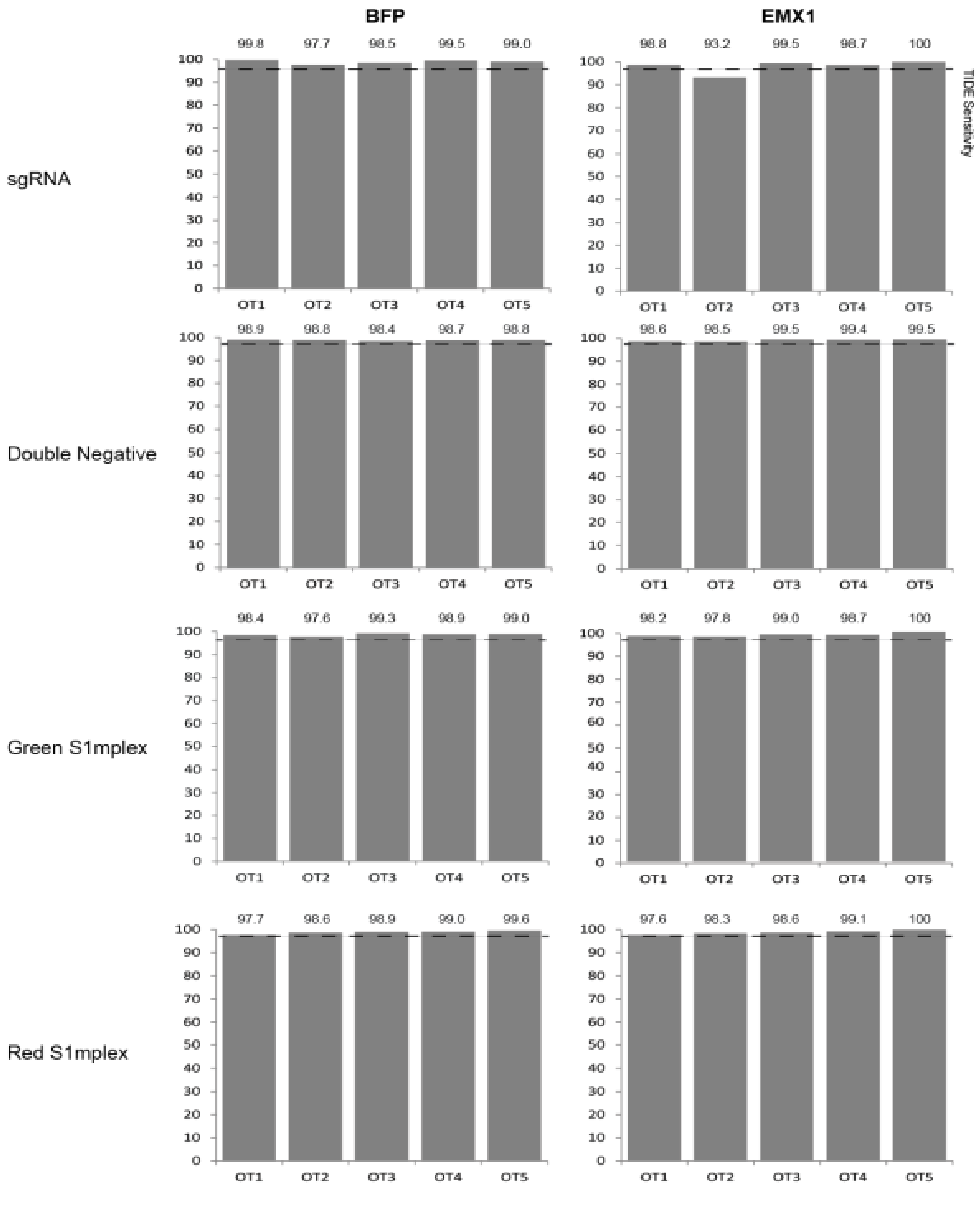

Supplementary Figure 7. Off-target analysis of sorted S1mplex populations.
Off-target analysis using TIDE software at the top 5 predicted off-target sites within the human genome at the *BFP* and *EMX1* loci. Y axis indicates the percentage of cells with 0 mismatches from the parental sequence (perfect matches in sequencing reads). None of the sorted S1mplex populations showed off-target effects above the limit of detection. The unsorted sgRNA RNP population had a small proportion of cells that may have been edited at OT-2 of the *EMX1* off-target sites.

Supplementary Table 1 Primers used to create sgRNA and S1m-sgRNA variants.

S1m Construct Name	Sequence (5’ to 3’)
S1m_StemLoop1_F	GTTTAAGAGCTATGCTGCGAATACGAGATGCGGCCGCCGACCAGAATCATGCAAGTGCGT AAGATAGTCGCGGGTCGGCGGCCGCATCTCGTATTC
S1m_StemLoop1_R	AAAAGCACCGACTCGGTGCCACTTTTTCAAGTTGATAACGGACTAGCCTTATTTAAACTT GCTATGCTGCGAATACGAGCCGCCGACCCG
S1m_StemLoop2_F	GTTTAAGAGCTATGCTGGAAACAGCATAGCAAGTTTAAATAAGGCTAGTCCGTTATCAAC TTCGAATACGAGATGCGGCCGCCGACCAGA
S1m_StemLoop2_R	AAAAAAAGCACCGACTCGGTGCCACTTTTTCCGAATACGAGatgcggCCGCCGACCCGCG ACTATCTTACGCACTTGCATGATTCTGGTCGGCGGC
S1m_StemLoop3_F	GTTTAAGAGCTATGCTGGAAACAGCATAGCAAGTTTAAATAAGGCTAGTCCGTTATCAAC TTGAAAAAGTGGCACCGAGTCGGTGCCGAA
S1m_StemLoop3_R	AAAAAAACGAATACGAGATGCGGCCGCCGACCCGCGACTATCTTACGCACTTGCATGATT CTGGTCGGCGGCCGCATCTCGTATTCGGCACCGACT
S1m1 Forward	TTAATACGACTCACTATAGGNNNNNNNNNNNNNNNNNNNNGTTTAAGAGCTATGCTGCGA
S1m2_3 Forward	TTAATACGACTCACTATAGGNNNNNNNNNNNNNNNNNNNNGTTTAAGAGCTATGCTGGAA
RNATracR	AAAAGCACCGACTCGGTGCC
S1m3_Reverse	AAAACGAATACGAGCCGCCG

Supplementary Table 2 Protospacer and respective PAMs used for genomic targeting.

sgRNA Name	Sequence (5' to 3')	PAM
BFP (BFP → GFP)	GCTGAAGCACTGCACGCCAT	GGG
EMX1 (EMX1_21)	GTCACCTCCAATGACTAGGG	TGG
mCherry (mCherry_15)	GGAGCCGTACATGAACTGAG	GGG
GAA ΔT	CTCGTTGTCCAGGTAGGCCC	GGG

Supplementary Table 3 Forward and reverse primers for genomic loci.

Genomic Primer	Forward (5' to 3')	Reverse (5' to 3')
EMX1	CCATCCCCTTCTGTGAATGT	GGAGATTGGAGACACGGAGA
EMX1 Symmetric	TCCACCTTGGCTTGGCTTTG	CCCTCCACCAGTACCCCAC
mCherry Interior	AAGGGCGAGGAGGATAACATGG	TTGTACAGCTCGTCCATGCCG
EMX1 Insertion	CCAATGACAAGCTTGCTAGC	GGAGATTGGAGACACGGAGA
GAA ΔT	AGCTGCTCATTGACCTCCAG	CAATCCACATGCCGTCGAAG

Supplementary Table 4 ssODNs used to direct HDR after DSB formation.

ssODN Donor	Sequence (5′ to 3′)
BFP → GFP NT	TCATGTGGTCGGGGTAGCGGCTGAAGCACTGCACGCCATGGGTCAGGGTGGTCACGAGGGTGGGCCAGGGCACCGGCAGCTTGCCGGTGGTGCAGATGAA
BFP → GFP 5PCBio NT	5Biotin/TCATGTGGTCGGGGTAGCGGCTGAAGCACTGCACGCCATGGGTCAGGGTGGTCACGAGGGTGGGCCAGGGCACCGGCAGCTTGCCGGTGGTGCAGATGAA
EMX1 NT	AAGCAGCACTCTGCCCTCGTGGGTTTGTGGTTGCCCACCGCTAGCAAGCTTGTCATTGGAGGTGACATCGATGTCCTCCCCATTGGCCTG
EMX1 5PCBio NT	5Biotin/AAGCAGCACTCTGCCCTCGTGGGTTTGTGGTTGCCCACCGCTAGCAAGCTTGTCATTGGAGGTGACATCGATGTCCTCCCCATTGGCCTG
3′ BFP → GFP -30+67 Antisense	GGCATGGCGGACTTGAAGAAGTCGTGCTGCTTCATGTGGTCGGGGTAGCGGCTGAAGCACTGCACGCCGTACGTCAGGGTGGTCACGAGGGTGGGCC/3Biotin
5′ BFP → GFP -30+67 Antisense	5Biotin/GGCATGGCGGACTTGAAGAAGTCGTGCTGCTTCATGTGGTCGGGGTAGCGGCTGAAGCACTGCACGCCGTACGTCAGGGTGGTCACGAGGGTGGGCC
3′ BFP → GFP -30+67 Sense	GGCCCACCCTCGTGACCACCCTGACGTACGGCGTGCAGTGCTTCAGCCGCTACCCCGACCACATGAAGCAGCACGACTTCTTCAAGTCCGCCATGCC/3Biotin
5′ BFP → GFP -30+67 Sense	5Biotin/GGCCCACCCTCGTGACCACCCTGACGTACGGCGTGCAGTGCTTCAGCCGCTACCCCGACCACATGAAGCAGCACGACTTCTTCAAGTCCGCCATGCC
3′ BFP → GFP -67+30 Antisense	GGTCGGGGTAGCGGCTGAAGCACTGCACGCCGTACGTCAGGGTGGTCACGAGGGTGGGCCAGGGCACCGGCAGCTTGCCGGTGGTGCAGATGAACTT/3Biotin
5′ BFP → GFP -67+30 Antisense	5Biotin/GGTCGGGGTAGCGGCTGAAGCACTGCACGCCGTACGTCAGGGTGGTCACGAGGGTGGGCCAGGGCACCGGCAGCTTGCCGGTGGTGCAGATGAACTT
3′ BFP → GFP -67+30 Sense	AAGTTCATCTGCACCACCGGCAAGCTGCCGGTGCCCTGGCCCACCCTCGTGACCACCCTGACGTACGGCGTGCAGTGCTTCAGCCGCTACCCCGACC/3Biotin
5′ BFP → GFP -67+30 Sense	5Biotin/AAGTTCATCTGCACCACCGGCAAGCTGCCGGTGCCCTGGCCCACCCTCGTGACCACCCTGACGTACGGCGTGCAGTGCTTCAGCCGCTACCCCGACC
3′ GAA Insert T -34+66 PAM	CTCCCCACTGCAGCCTCTCGTTGTCCAGGTATGGCCCGGGTCCACTGCCTTCCCCGACTTCACCAACCCCACAGCCCTGGCCTGGTGGGAGGACATGGTG/3Biotin
5′ GAA Insert T -34+66 PAM	5Biotin/CTCCCCACTGCAGCCTCTCGTTGTCCAGGTATGGCCCGGGTCCACTGCCTTCCCCGACTTCACCAACCCCACAGCCCTGGCCTGGTGGGAGGACATGGTG
3′ GAA Insert T -34+66 NonPAM	CACCATGTCCTCCCACCAGGCCAGGGCTGTGGGGTTGGTGAAGTCGGGGAAGGCAGTGGACCCGGGCCATACCTGGACAACGAGAGGCTGCAGTGGGGAG/3Biotin
5′ GAA Insert T -34+66 NonPAM	5Biotin/CACCATGTCCTCCCACCAGGCCAGGGCTGTGGGGTTGGTGAAGTCGGGGAAGGCAGTGGACCCGGGCCATACCTGGACAACGAGAGGCTGCAGTGGGGAG

Supplementary Table 5 Off target sequences and corresponding genomic locus for each sgRNA used.

sgRNA Target Sequence	Off-Target Sequence		PAM	Locus
BFP→ GFP<br>GCTGAAGCACTGCACGCCAT	OT1	GCAGAAGCACTGCAAGCCAT	CAG	chr17:+39786906
	OT2	TCTGAAGTGCTGCACGCCAT	CAG	chr2:-238397265
	OT3	GTGGAAGCACTGCAAGCCAT	TGG	chr7:-11228464
	OT4	GGTGGAGCAGGGCACGCCAT	CAG	chr9:+109114765
	OT5	GAAGAAGCACTGCACCCCAT	CAG	chr13:-75660548
EMX1<br>GTCACCTCCAATGACTAGGG	OT1	AGGACCACCAATGACTAGGG	CAG	chr3:-64303990
	OT2	ACCACCTGTAATGACTAGGG	TAG	chr4:-149749778
	OT3	GGAGCCTCCAGTGACTAGGG	GAG	chr17:-38423030
	OT4	GTGAACTACAGTGACTAGGG	TGG	chr8:+112210096
	OT5	CTGGCCTCCAAAGACTAGGG	GAG	chr15:-75011931

Mismatches from protospacer are labelled in red

Supplementary Table 6 Forward and reverse primers used to amplify off-target genomic loci.

Off-Target Primer	Forward (5’ to 3’)	Reverse (5’ to 3’)
BFP OT1	TTTCCTAGCAAGCAGACTCAGA	AGCTGTCCTTTGTCCCATTGA
BFP OT2	TCTCCATGCCCTCCTTTCCAT	GGATGTAGTCCATGATCTTCCCC
BFP OT3	TCCCAGAATGTGAAAGTGGAGG	CTGTGGGCTTTCCTCAGCTC
BFP OT4	GCTGACTAACGTCCACTGCT	TGGACCTATGTTTTTCTTCGTCAC
BFP OT5	AAAGTCTGTGGCCTTGTGAGA	AACCCTACCCCCTACCTGAA
EMX1 OT1	TTCCCCAGGTAGTTGCTGTTC	TCTGCACATGTCCCAACTGTC
EMX1 OT2	ATCCGTACCTAACCATGACCC	GCACAGATCTTGGTGGCTTT
EMX1 OT3	GGCTGGGTTTCCCAAACGTA	CAAACTGCTGTGTTGGGTGG
EMX1 OT4	ACTTGGAAGGGTCCACACAA	CCTTGAATAGAGCATTTTTCCCCA
EMX1 OT5	TCCTACCCTTGGATGGGGTT	GGGCTACACGGTCCCTAAAG

Supplementary Table 7 Next generation sequencing primers including adapters and barcode around DSB site.

NGS Primer	Sequence (5' to 3')
BFP→ GFP F	TCATCTGCACCACCGGCAAG
BFP→ GFP F1	AATGATACGGCGACCACCGAGATCTACACTATAGCCTACACTCTTTCCCTACACGACGCTCTTCCGATCTTCATCTGCACCACCGGCAAG
BFP→ GFP F2	AATGATACGGCGACCACCGAGATCTACACATAGAGGCACACTCTTTCCCTACACGACGCTCTTCCGATCTTCATCTGCACCACCGGCAAG
BFP→ GFP F3	AATGATACGGCGACCACCGAGATCTACACCCTATCCTACACTCTTTCCCTACACGACGCTCTTCCGATCTTCATCTGCACCACCGGCAAG
BFP→ GFP R	GAAGTCGTGCTGCTTCATGTGG
BFP→ GFP R1	CAAGCAGAAGACGGCATACGAGATCTGAAGCTGTGACTGGAGTTCAGACGTGTGCTCTTCCGATCGAAGTCGTGCTGCTTCATGTGG
BFP→ GFP R2	CAAGCAGAAGACGGCATACGAGATTAATGCGCGTGACTGGAGTTCAGACGTGTGCTCTTCCGATCGAAGTCGTGCTGCTTCATGTGG
EMX1 F	CTCCCATCACATCAACCGGTGG
EMX1 F1	AATGATACGGCGACCACCGAGATCTACACTATAGCCTACACTCTTTCCCTACACGACGCTCTTCCGATCTCTCCCATCACATCAACCGGTGG
EMX1 F2	AATGATACGGCGACCACCGAGATCTACACATAGAGGCACACTCTTTCCCTACACGACGCTCTTCCGATCTCTCCCATCACATCAACCGGTGG
EMX1 F3	AATGATACGGCGACCACCGAGATCTACACCCTATCCTACACTCTTTCCCTACACGACGCTCTTCCGATCTCTCCCATCACATCAACCGGTGG
EMX1 R	CACTCTGCCCTCGTGGGTTT
EMX1 R1	CAAGCAGAAGACGGCATACGAGATCGCTCATTGTGACTGGAGTTCAGACGTGTGCTCTTCCGATCCACTCTGCCCTCGTGGGTTT
EMX1 R2	CAAGCAGAAGACGGCATACGAGATGAGATTCCGTGACTGGAGTTCAGACGTGTGCTCTTCCGATCCACTCTGCCCTCGTGGGTTT
GAA F	AGTGGGGCTTCCATGCAG
GAA R	GGTTGGTGAAGTCGGGGAAG

Supplementary Table 8 Absolute levels of editing in this study and prior studies to date.

**Experiment**	**Cell type**	**Gene**	**# of base pair changes**	**% of HDR** (absolute percent of deep sequencing reads)	**% of NHEJ** (absolute percent of deep sequencing reads)	**Reference**
Figure 3, averaged	HEK	*BFP, EMX1*	3, 12	0.53%	0.16 %	This study
Figure 3, averaged	hPSC	*BFP, EMX1*	18	0.34%	1.89%	This study
Figure 4, averaged	hPSC	*BFP, GAA*	2-3	2.0%	0.58%	This study
Figure 6, averaged	HEK	*BFP and EMX1*	3 and 12	0.34%	0.14%	This study
Averaged across experiments involving HDR as assayed by deep sequencing (Figs. 3, 4, 6)	HEK, hPSC	*BFP, GAA, EMX1*	2 to 18	1.6%	0.67%	This study
Small base pair changes	HEK, hPSC	*BFP, GAA*	<5	1.8%	0.75%	This study
HDR as assayed by Sanger sequencing of enriched clones (Fig. 6b)	HEK	*BFP*	3	24%	24%	This study
Figure 1	hPSC	*SCN8A, SCN1B, CHD2, PCDH19, HPRT1, SMC1A*	N/A	N/A	1-17.5%	A. M. Tidball et. al. *Stem Cell Reports*, 2017
Extended data Figure 1f	hPSC	*MYBPC3*	4	1.5%	3.8%	H. Ma et. al. *Nature*, 548, 413–419, 2017
Extended data Table 1	hPSC	*APP, PSEN1*	2	0.3-6.0%	Not reported	Paquet, D. *et al. Nature* **533,** 125–129 (2016).
Figure 3a	hPSC	*CCR5*	2	0.2-1.6%	1-1.8%	Yang, L. *et al. Nucl. Acids Res.* (2013). doi:10.1093/nar/gkt555
Table S1	hPSC	*AKT2*	1	6.4%*	Not reported	Ding, Q. *et al. Cell Stem Cell* **12,** 393–394 (2013).
Figure 2f	hPSC	*SOD1*	1	0.35-3.1%	27-32%	Yu, C. *et al. Cell Stem Cell* **16,** 142–147 (2015).

Figure 5	HEK293T	*RBM20, ATP7B*	1	0.09-0.6%	3-9%	Miyaoka, Y. *et al. Scientific Reports* **6,** srep23549 (2016).
Figure 5	hPSC	*RBM20*	1	0.005-0.1%	1-10%	Miyaoka, Y. *et al. Scientific Reports* **6,** srep23549 (2016).
Figure 2c, Supplementary Table 12 before drug selection	HCT116	*ERCC3*	2	0.3-5.2%	7.1-12.6%	Smurnyy, Y. et al. Nat Chem Biol 10, 623–625 (2014).
Figure 2a, unexpanded cells	HSPC	*HBB*	1	6-11%	10-30%	DeWitt, M. A. *et al. Science Translational Medicine* **8,** 360ra134- (2016).
Figure 4c	hPSC	*EGFP*	2	2.5-4.5%**	30-60%**	Howden, S. E. *et al. Stem Cell Reports* doi:10.1016/j.stemcr.2016.07.001
Supplemental Table S2	HSPC	*CD45*	2	24%	16%***	Gundry, M. C. *et al. Cell Rep* 17, 1453–1461, 2016
Figure 3c	HEK	*BFP*	3	9-63%	25%-90%	Richardson, C. D., Ray, G. J., DeWitt, M. A., Curie, G. L. & Corn, J. E. *Nat Biotech* 34, 339–344, 2016
Figure S6, 7	HEK	*EMX1*	4	43-66%	2-31%****	Richardson, C. D., Ray, G. J., DeWitt, M. A., Curie, G. L. & Corn, J. E. *Nat Biotech* 34, 339–344, 2016
Figure 3	HEK	*EMX1*	12	0-6%*****	Not reported	Lin, S. et al. eLife 2014;3:e04766
Figure 4	hPSC	*EMX1*	12	0-1.6%	3-44%	Lin, S. *et al.* eLife 2014;3:e04766

*enriched by flow cytometry for Cas9-GFP expressing cells; 10 of 94 clones with monoallelic mutation introduced and 1 of 94 clones with biallelic HDR editing, resulting in 12 out of 188 alleles = 6.4%

** with plasmid HDR donors; assayed by flow cytometry

***Only the most common indels are reported. Reported frequencies for all alleles add up to 85% in this table.

**** ssODN most similar to work involved in this paper induced 2-9% HDR measured by Restriction Fragment Length Polymorphism (RFLP) assay.

***** ssODN similar to work in the paper was below limit of detection using Restriction Fragment Length Polymorphism (RFLP) assay.

Prior studies were selected based on a comparable number of base pair changes within the ssODN template and use of deep or Sanger sequencing of the edited locus. Deep sequencing assays were performed shortly after RNP delivery without significant selection, while Sanger sequencing occurred after clonal isolation and expansion without drug selection.

Supplementary Table 9 Absolute levels of editing for each experimental replicate assayed by deep sequencing.

Experiment	Condition	Cell Line	Locus	Number of base pair changes	HDR1	HDR2	HDR3	NHEJ1	NHEJ2	NHEJ3
Figure 3, also Supplementary Figure 4	ssODN-S1m-sgRNA-1 S1mplex	hPSC	BFP	3	0.49%			0.43%		
	ssODN-S1m- sgRNA-2 S1mplex	hPSC	BFP	3	0.76%			0.68%		
	ssODN-S1m- sgRNA-3 S1mplex	hPSC	BFP	3	0.30%			0.35%		
	Standard sgRNA ssODN	hPSC	BFP	3	1.3%			3.9%		
	Standard sgRNA ssODN	HEK	BFP	3	0.52%			2.2%		
	ssODN-S1mplex	HEK	BFP	3	0.99%			0.23%		
	Standard sgRNA ssODN	HEK	EMX1	12	1.0%			3.9%		
	ssODN-S1mplex	HEK	EMX1	12	0.07%			0.09%		
	Standard sgRNA ssODN	hPSC	BFP	18	0.045%			11%		
	sgRNA Bio-ssODN	hPSC	BFP	18	0.002%			9.8%		
	S1m Bio-ssODN (-SA)	hPSC	BFP	18	0.009%			1.3%		
	ssODN-S1mplex	hPSC	BFP	18	0.079%			2.0%		
	S1m Bio-ssODN (-SA)	hPSC	EMX1	18	0.001%			1.8%		
	ssODN-S1mplex	hPSC	EMX1	18	0.052%			6.0%		
Figure 4	5' -67 NonPAM	hPSC	BFP	3	1.0%	1.1%	3.6%	0.51%	0.22%	0.94%
	3'-67 NonPAM	hPSC	BFP	3	1.0%	0.97%	5.9%	0.18%	0.15%	1.7%
	5' -30 NonPAM	hPSC	BFP	3	1.1%	1.2%	3.1%	0.11%	0.30%	1.4%
	3' -30 NonPAM	hPSC	BFP	3	1.0%	1.3%	3.4%	0.31%	0.44%	1.5%
	5' -67 PAM	hPSC	BFP	3	1.3%	1.1%	2.8%	0.37%	0.13%	1.4%
	3' -67 PAM	hPSC	BFP	3	1.0%	1.2%	5.0%	0.34%	0.34%	2.0%
	5' -30 PAM	hPSC	BFP	3	1.1%	1.1%	6.6%	0.27%	0.25%	1.4%
	3' -30 PAM	hPSC	BFP	3	1.0%	1.0%	3.4%	0.20%	0.24%	1.4%
	RNP Control	hPSC	BFP	3	3.1%	1.1%	1.4%	2.9%	2.4%	4.6%
	RNP Control	hPSC	GAA	2	1.7%	2.7%		2.2%	3.7%	

Experiment	Condition	Cell Line	Locus	Number of base pair changes	HDR1	HDR2	HDR3	NHEJ1	NHEJ2	NHEJ3
	3' -34 PAM	hPSC	GAA	2	1.2%	3.8%		0.18%	0.46%	
	5' -34 PAM	hPSC	GAA	2	1.4%	2.6%		0.40%	0.37%	
	3' -34 NonPAM	hPSC	GAA	2	1.1%	1.0%		0.17%	0.33%	
	5' -34 NonPAM	hPSC	GAA	2	1.1%	1.1%		0.18%	0.27%	
**Figure 6d,** also Supplementary Figure 6b *Multiplexed editing* *for both EMX and BFP*	Double Negative	HEK	EMX1	12	0.02%			0.04%		
	Green+	HEK	EMX1	12	0.15%			0.36%		
	Red+	HEK	EMX1	12	0.02%			0.04%		
	Double Positive	HEK	EMX1	12	0.10%			0.13%		
	Double Negative	HEK	BFP	3	0.29%			0.05%		
	Green+	HEK	BFP	3	0.11%			0.02%		
	Red+	HEK	BFP	3	0.52%			0.01%		
	Double Positive	HEK	BFP	3	0.57%			0.04%		

Analysis of HDR and NHEJ rates following deep sequencing in each experimental condition and replicate presented in this work.

All deep sequencing data and Sanger sequencing traces from PCR amplicons are available at Bioproject PRJNA381066.

SUPPLEMENTARY NOTE 1

In parallel with the deep sequencing assays (**Supplementary Table 9)**, flow cytometry and PCR-based assays were used to assay for precise and imprecise gene editing with the S1mplexes. For the ssODN-S1mplexes, we found that ssODN-S1mplexes significantly decreased imprecise NHEJ gene editing as measured by loss of BFP (**Supplemental Figure 4e**), consistent with the results from the sgRNA variant editing experiments (**Supplementary Figure 3a**). At the same time, ssODN-S1mplexes produced robust levels of precise editing as indicated by the gain of GFP (**Supplementary Figure 4d**). The ratio of precisely-edited GFP cells to improperly-edited non-fluorescent cells increased 5-fold with ssODN-S1mplexes (**Supplementary Figure 4c**). Importantly, these increases were abrogated by omitting any of the critical components of the complex (either S1m aptamer, streptavidin or biotin) in the formulation. Compared to the approximately 2-fold decrease in NHEJ editing by utilizing a S1m-sgRNA (**Supplementary Figure 3a**), the 5-fold increase in the precise: imprecise ratio could not be attributed solely to the presences of a S1m aptamer.

For the fluorescent ssODN-S1mplexes, we also analyzed each sorted population using conventional flow cytometry and PCR-based methods. At the *BFP* locus each ssODN-S1mplex sorted population had similar levels of BFP-negative cells as the control (**Supplementary Figure 6d**). However, when we analyzed GFP fluorescence gain (indicating precise editing via HDR), cells that were positive for complexes targeted against the *BFP* locus (Red+ and the double positive fractions) had increased levels of precise editing in comparison to the other two sorted cell populations (**Supplementary Figure 6c**). It was observed that the Red+ population had 3.5-fold higher precise-to-imprecise editing ratio while the double positive had a 4.5-fold increase over the double negative population, respectively (**Supplementary Figure 6c**). As seen from deep sequencing data (**Supplementary Figure 6b**), these ratios are likely underrepresented in flow cytometry assays, due to the presence of BFP-negative cells in the starting population (4.08% in **Supplementary Figure 6d**, right). We next tested for the insertion of the 12 nt sequence into the *EMX1* locus by creating a PCR primer that was specific to only the insert. Populations specific for *EMX1* editing (Green+ and double positive fractions) displayed strong bands at the expected size. The Green+ population had a 3-fold increase in intensity when compared background levels, while the double positive population increased HDR 24-fold (**Supplementary Figure 6c**, left). Overall, the trend of increased precise editing relative to imprecise editing with the flow cytometry and PCR assays are consistent with the deep sequencing results in **Figures 3** and **6 and displayed in Supplementary Table 9**. Deep sequencing of the target locus, particularly when using ssODNs, allows for faithful determination of precise repair across the entire homology region, which is difficult to determine with the flow cytometry of reporter gene disruption (e.g., BFP loss/GFP gain in **Supplementary Figure 4** and **6** and with traffic light reporters[1]) and PCR-based assays involving

insertion of unique sequences (e.g., unique primer as in **Supplementary Figure 6** or restriction site[2]). The use of large homology arms, such as in plasmid-based and virally-delivered donors used previously[1,3], that extend past the region of interest may contain also mismatches that remain undetected even after HDR. Further, while high targeted insertion rates have been reported with plasmid-based transfection methods[1,4], these reports focus on selected cells from the transfected cell population and do not quantify the levels of NHEJ of the entire cell population. For RNP delivery with ssODNs to human cells[3,5], deep sequencing results have at best generated approximately one precise edit to each imprecise edit detected per million reads of the full transfected population.

SUPPLEMENTARY REFERENCES

1. Chu, V. T. *et al.* Increasing the efficiency of homology-directed repair for CRISPR-Cas9-induced precise gene editing in mammalian cells. *Nat. Biotechnol.* **33,** 543–548 (2015).

2. Ran, F. A. *et al.* Genome engineering using the CRISPR-Cas9 system. *Nat. Protoc.* **8,** 2281–2308 (2013).

3. Dever, D. P. *et al.* CRISPR/Cas9 β-globin gene targeting in human haematopoietic stem cells. *Nature* **539,** 384–389 (2016).

4. Paquet, D. *et al.* Efficient introduction of specific homozygous and heterozygous mutations using CRISPR/Cas9. *Nature* **533,** 125–129 (2016).

5. DeWitt, M. A. *et al.* Selection-free genome editing of the sickle mutation in human adult hematopoietic stem/progenitor cells. *Sci. Transl. Med.* **8,** 360ra134-360ra134 (2016).

6. Song, J. *et al.* RS-1 enhances CRISPR/Cas9- and TALEN-mediated knock-in efficiency. *Nat. Commun.* **7,** 10548 (2016).

7. Liang, X., Potter, J., Kumar, S., Ravinder, N. & Chesnut, J. D. Enhanced CRISPR/Cas9-mediated precise genome editing by improved design and delivery of gRNA, Cas9 nuclease, and donor DNA. *J. Biotechnol.* **241,** 136–146 (2017).
